# A New Fluorescence-Based Method Identifies Protein Phosphatases Regulating Lipid Droplet Metabolism

**DOI:** 10.1371/journal.pone.0013692

**Published:** 2010-10-28

**Authors:** Bruno L. Bozaquel-Morais, Juliana B. Madeira, Clarissa M. Maya-Monteiro, Claudio A. Masuda, Mónica Montero-Lomeli

**Affiliations:** 1 Instituto de Bioquímica Médica, Programa de Biologia Molecular e Biotecnologia, Universidade Federal do Rio de Janeiro, Rio de Janeiro, Rio de Janeiro, Brazil; 2 Laboratório de Imunofarmacologia, Instituto Oswaldo Cruz, Fundação Oswaldo Cruz, Manguinhos, Rio de Janeiro, Rio de Janeiro, Brazil; CNRS UMR6543, Université de Nice, Sophia Antipolis, France

## Abstract

In virtually every cell, neutral lipids are stored in cytoplasmic structures called lipid droplets (LDs) and also referred to as lipid bodies or lipid particles. We developed a rapid high-throughput assay based on the recovery of quenched BODIPY-fluorescence that allows to quantify lipid droplets. The method was validated by monitoring lipid droplet turnover during growth of a yeast culture and by screening a group of strains deleted in genes known to be involved in lipid metabolism. In both tests, the fluorimetric assay showed high sensitivity and good agreement with previously reported data using microscopy. We used this method for high-throughput identification of protein phosphatases involved in lipid droplet metabolism. From 65 yeast knockout strains encoding protein phosphatases and its regulatory subunits, 13 strains revealed to have abnormal levels of lipid droplets, 10 of them having high lipid droplet content. Strains deleted for type I protein phosphatases and related regulators (*ppz2*, *gac1*, *bni4*), type 2A phosphatase and its related regulator (*pph21* and *sap185*), type 2C protein phosphatases (*ptc1*, *ptc4*, *ptc7*) and dual phosphatases (*pps1*, *msg5*) were catalogued as high-lipid droplet content strains. Only *reg1*, a targeting subunit of the type 1 phosphatase Glc7p, and members of the nutrient-sensitive TOR pathway (*sit4* and the regulatory subunit *sap190*) were catalogued as low-lipid droplet content strains, which were studied further. We show that Snf1, the homologue of the mammalian AMP-activated kinase, is constitutively phosphorylated (hyperactive) in *sit4* and *sap190* strains leading to a reduction of acetyl-CoA carboxylase activity. In conclusion, our fast and highly sensitive method permitted us to catalogue protein phosphatases involved in the regulation of LD metabolism and present evidence indicating that the TOR pathway and the SNF1/AMPK pathway are connected through the Sit4p-Sap190p pair in the control of lipid droplet biogenesis.

## Introduction

In virtually every cell, neutral lipids are stored in cytoplasmic structures called lipid droplets (LDs) (rev. [1]). In the budding yeast *S. cerevisiae*, these structures contain a neutral lipid core composed of triacylglycerols (TAGs) and sterol esters (SEs) in a 1∶1 ratio [2]. This core is delimited by a phospholipid monolayer associated with a diversity of proteins, which have structural functions and also show enzymatic activity [3], [4]. LDs are thought to originate in the endoplasmic reticulum [5], [6]. It is thought that as TAG and SE are synthesized, they accumulate between the internal and external endoplasmic reticulum membrane leaflets until LDs bud from the endoplasmic reticulum [7]. This hypothesis is reinforced by the fact that the enzymes that contribute to the esterification of fatty acids (FAs) and sterols (STEs) e.g., diacylglycerol acyltransferases Dga1p [8] and Lro1p [9], and acyl-CoA:sterol acyltransferases, Are1p and Are2p are localized to the endoplasmic reticulum [10]. Among these enzymes, only Dga1p is found in LDs [11]. Regarding the utilization of stored lipids, five enzymes responsible for the mobilization of stored lipids are localized to the LD. Three of these enzymes are TAG lipases – Tgl3p, Tgl4p and Tgl5p [12], [13] – and two are SE hydrolases – Yeh1p and Tgl1p [14]. Remarkably, the major enzyme contributing to SE hydrolase activity (Yeh2p) is localized at the plasma membrane [15]. This raises the question of whether the LD is more than a mere neutral lipid deposit. In fact, equilibrium between mobilization and LD synthesis seems to be important in various aspects of metabolism, such as cell cycle progression, that requires lipolysis [16], membrane trafficking and “lipid buffering” to regulate levels of free FA [17]. Once synthesized, free FAs and free STEs would represent a lipotoxic threat [18] if not converted by esterification into more biologically inert lipids, such as TAG and SEs, and then stored in LDs. In yeast, deletion of the acyltransferases that esterify fatty acids into TAGs and SEs lead to high sensitivity to unsaturated fatty acids [19]. In mammals, LDs control the synthesis and secretion of inflammatory mediators [20] and are implicated in virus propagation [21]. Different stimuli and signaling pathways are involved in the biogenesis of LDs; for example, in macrophages, leptin-induced LD formation is mediated by phosphatidylinositol 3-kinase (PI3-K) and the mammalian target of rapamycin (mTOR) pathways [22].

In yeast, LDs are quite dynamic organelles. It was observed that they are consumed as a response to a proliferative stimulus, such as the addition of rich medium to stationary cells. During the lag phase of growth [23], when nutrients are abundant, lipid mobilization provides construction blocks for the synthesis of more complex lipids such as phospholipids. When cells enter the exponential phase of growth, LDs are re-synthesized [23]. Nevertheless, little is known about the regulators and pathways driving these dynamics. It is known that Snf1, a homolog to mammalian AMP-activated kinase (AMPK), is a negative regulator of fatty acid synthesis and, once phosphorylated, mediates acetyl-CoA carboxylase phosphorylation and subsequent inhibition [24], [25]. In order to determine the role of protein phosphorylation in the regulation of LD dynamics, we screened yeast mutant strains in which protein phosphatases (including catalytic and regulatory subunits) had been knocked out (no phosphatase has been reported to regulate lipid droplet metabolism). Since the techniques used in the study of lipids are laborious and require time-consuming microscopic analysis, our group developed a new fluorimetric method suitable for high-throughput experiments. Nile red is an excellent fluorescent probe for detection of intracellular lipid droplets by fluorescence microscopy and flow cytofluorometry [26]–[28]. BODIPY® (4,4-difluoro-3a,4a,-diaza-s-indacene) has also been used in LD microscopic studies, and has previously been used for a screening of a yeast deletion mutant collection for LD abnormalities [29]. Although both molecules preferentially stain LDs, the latter seems to be more specific [26]. Based on BODIPY fluorescence, we were able to rapidly evaluate LD content without lipid extraction or microscopy analysis. Our results showed that this method is suitable for following LD dynamics during yeast culture growth and can be employed to screen a large number of mutants.

Using our new approach, we were able to identify 13 (out of 65) phosphatase knockout strains that presented abnormal LD levels. Among these, we further studied Sit4, a Ser/Thr protein phosphatase, as a positive regulator of LD metabolism. We propose that Sit4p, in association with its regulatory subunit Sap190p, regulates lipid metabolism by controlling the phosphorylation status of Snf1 kinase. In fact, the snf1-null strain showed increased LD levels, confirming unpublished data from Kohlwein and coworkers [30], while sit4- or sap190-null strains exhibited the opposite phenotype, which is consistent with the hyperphosphorylation of Snf1p observed in these strains.

## Materials and Methods

### Reagents

BODIPY® 493/503 (referred to here as BODIPY) was purchased from Invitrogen, prepared as a 10 mM stock solution in DMSO and kept at −70°C. Soraphen A was a gift from Rolf Müller (Helmoltz-Zentrum für Infektionsforschung) and kept as a 0.1 mg/ml stock solution in 10% methanol. All other reagents were from Sigma-Aldrich (St. Louis, MO).

### Strains

*Saccharomyces cerevisiae* strain *BY4741* (MATa *his3Δ1 leu2Δ0 met15Δ0 ura3Δ0*) and *BY4741*- MATa gene deletion library (Open Biosystems). *BY4742* (MATα *his3Δ1 leu2Δ0 lys2Δ0 ura3Δ0*), *dga1lro1* (MATα *his3Δ1 leu2Δ0 lys2Δ0 ura3Δ0 dga1::KanMX lro1::KanMX*) and *are1are2* (MATα *his3Δ1 leu2Δ0 lys2Δ0 ura3Δ0 are1::KanMX are2::KanMX*) were kindly provided by Sepp D. Kohlwein (University of Graz, Austria).

### Yeast growth and cell fixation

*Saccharomyces cerevisiae* strains were grown in YPD liquid-rich medium (1% yeast extract, 2% peptone and 2% glucose) at 30°C. For determination of LD content, aliquots were withdrawn during growth and cells were immediately fixed by adding 1/9 volume of formaldehyde (final concentration 3.7% v/v). After incubation for 15 min at 25°C, cells were collected by centrifugation (3000 rpm, 5 min) and washed once with distilled water. Cells were resuspended in water and adjusted to 5 O.D._600 nm/_ml and kept at 4°C until use. For use in high-throughput screening selected strains from the *BY4741*- MATa gene deletion library (Open Biosystems) were cultured overnight in YPD medium in 96-well plates. Afterwards they were transferred to fresh YPD medium with the aid of a 96 -Pin Replicator and grown at 30°C with shaking. Growth was monitored during 48 hours to ensure that all deleted strains, even those that have a slow-growth phenotype, had attained the stationary phase. The OD_600_ varied between 2.8–6.3. The mean growth was OD_600_ = 4.2±0.6. Each sample was then fixed by the addition of 22 µl of formaldehyde, incubated for 15 min at 25°C and washed with distilled water. Fixed cells were harvested and resuspended in 200 µl of distilled water.

### Liquid fluorescence recovery assay

**Figure 1:**
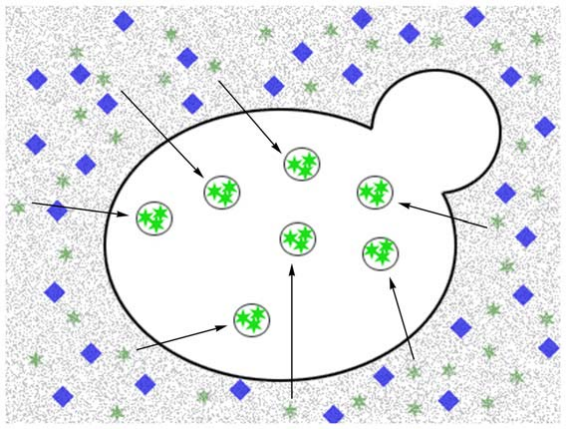
A schematic representation of the fluorescence recovery assay. Based on the high specificity of the fluorescent probe BODIPY 493/503 for lipid droplets, we designed a rapid fluorimetric method for the evaluation of lipid droplets inside yeast cells. With this method, BODIPY's fluorescence is quenched in solution and, when cells are added, fluorescence is recovered due to passive BODIPY dislocation from the medium to lipid droplets, from which the quencher is excluded.

LD content was determined by recovering the fluorescence of quenched BODIPY following the addition of fixed cells (Figure 1). For this purpose, 5 µl of a formaldehyde-fixed cell suspension was added to 200 µl of quenched reading buffer containing 5 µM BODIPY and 500 mM KI, all contained in a 96-well black-wall/clear-bottom plate (Costar). The settings for fluorescence were excitation 480 nm, emission 510 nm, cutoff filter at 495 nm, PMT automatic. Concomitant to each fluorescence reading, the quantity of cells was determined by measuring the absorbance at 600 nm. Each sample was read four times by adding subsequent 5-µl aliquots of a formaldehyde-fixed cell suspension to the same well. Reading blanks were acquired by incubating the reading buffer for 5 min at 30°C before the addition of cells. Data quality was analyzed by evaluating the linearity of measurements compared to cell number. From this curve, the relative fluorescence per number of cells was determined and denoted the lipid droplet index (LD index). If R-square <0.9, data were discarded (Figure S1 and Figure S2).

### Statistical analysis of high-throughput experiments

In order to classify strains as high lipid droplet content (*hlc*) or low lipid droplet content (*llc*) with respect to wild-type, LD indexes were compared as follows. For each independent experiment, the LD index of each individual strain was normalized to the mean LD index of all samples contained in the same plate. Then, the mean LD index from four independent experiments was compared to the mean LD index of the wild-type (WT) strain (n = 14). The F-test was performed to determine whether the variability in the data collected for each deletion strain was similar to that observed for the wild-type strain; a homoscedastic or heteroscedastic t-test was employed accordingly. Strains with values statistically different (p<0.05) were catalogued as *hlc* or *llc* strains when the normalized LD index was at least 20% higher or lower than that of the wild-type strain, respectively.

### Quantification of lipid droplets by fluorescence microscopy

Aliquots of 5 µl of cellular suspension (10 OD 600 nm) and 5 µl of 10 µM BODIPY were mounted onto a polylysine-treated glass slide. Microscopy was performed on Axio Observer.Z1 (Zeiss) or Olympus IX 81 using a 100x oil immersion objective. Images captured were analyzed using ImageJ software and total fluorescence was determined with the aid of the nucleus counter and cell counter function of the Wright Cell Imaging Facility (WCIF) plug-in collection. LDs were quantified in at least 50 cells.

### Protein homogenate preparation and western blot

Total protein homogenates were prepared using the rapid procedure described by Yaffe and Schatz [31] with minor modifications. Briefly, 0.25 M NaOH and 1% β-mercaptoethanol (final concentration) were added to culture samples, which were then incubated at room temperature for 10 min, followed by the addition of trichloroacetic acid to a final concentration of 6%. Protein was collected by centrifugation and the pellets were stored at −80°C until use. For western blot analysis, pellets were resuspended directly in electrophoresis Laemlli sample buffer to provide a 10-OD suspension. Aliquots of 10 µl of these extracts, containing 0.1-OD cells, were separated in 7.5% SDS-acrylamide gel using the Mini-Protean II (BioRad) and electrotransferred to Immobilon-P for 30 min at 18 V in 25 mM Tris, 192 mM glycine and 10% methanol, using a trans-blot semi-dry cell (BioRad). Membranes were treated with anti-phospho-AMPKα Thr172 (Cell Signaling) or anti-Snf1p (Santa Cruz) antibodies. Blots were detected using the chemiluminescence ECL Plus kit (GE Healthcare).

### Measurement of triacylglycerol

Cultures were grown in YPD medium, harvested by centrifugation at 3000 rpm for 5 minutes at room temperature and washed with distilled water. An aliquot of 15 OD cells was resuspended in 300 µl of extraction buffer (50 mM Tris-HCl, 0.3% Triton X-100, pH 7.5) and lysed with glass beads vortexing for 5 cycles of 30 seconds at 4°C. Lysed cells were separated and the glass beads were washed with 300 µl of extraction buffer. The total lysate was centrifuged at 3,000 rpm for ten minutes. Neutral lipids were extracted from 200 µl of the supernatant as described by Bligh & Dyer [32]. Briefly cells were incubated with shaking for 5 minutes after successive addition of 750 µl chloroform/methanol (1∶2), 250 µl chloroform and 250 µl distilled water. Tubes were centrifuged at 1,000 rpm for 5 minutes and the organic phase was dried under N_2_ vapor at room temperature. Neutral lipids were resuspended in 150 µl of extraction buffer. Triglycerides were measured with a clinical kit (Doles, Brazil) as indicated by the manufacturer. This kit is based on hydrolysis of triacylglycerol with lipase and further determination of glycerol.

### Soraphen A susceptibility tests

Drug susceptibilities were measured by both broth microdilution and spot assays. MICs for the strains were determined using the broth microdilution method. Spot assays were carried out as in [33]. Briefly, yeast strains were grown in YPD to stationary phase and inoculated at serial dilutions of 10^7^, 10^6^ and 10^5^ cells/ml onto YPD plus 2% agar with increasing concentrations of soraphen A and then incubated for two days at 30°C, as previously described [34]. For microdilution tests, cells from stationary culture were diluted to 10^5^ cells/ml in fresh YPD with increasing concentrations of soraphen A in a total volume of 200 µl and then incubated in 96-well microtiter plates at 30°C. Growth was recorded after 24 h by measuring absorbance at 600 nm. The IC_50_ was defined as the drug concentration that inhibited growth by 50% compared to growth of the drug-free controls.

## Results

### Liquid fluorescence recovery assay (LFR assay)

**Figure 2:**
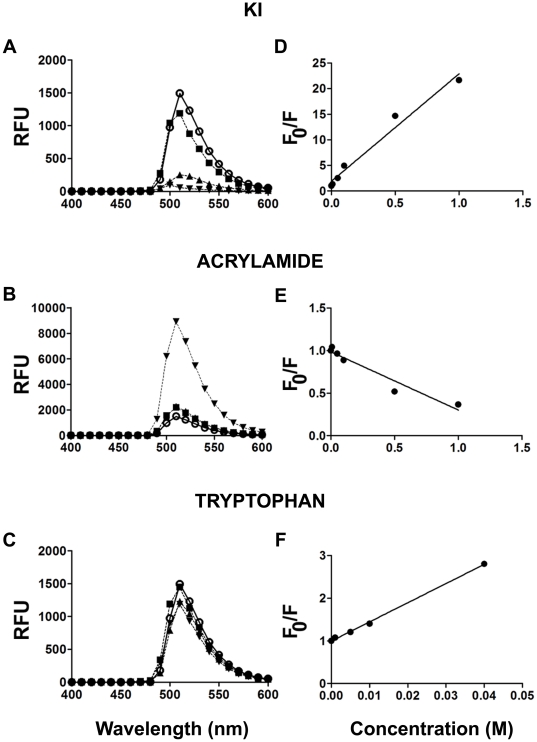
Quenchers of BODIPY fluorescence. *A and B*, Emission spectra (ex 485 nm) of a 5 µM aqueous solution of BODIPY in the presence of KI (A), acrylamide (B) and tryptophane (C). The concentrations of KI and acrylamide were: none (○), 0.01 M (▪), 0.1 M (▴) and 1 M (▾), Due to its reduced solubility in the conditions tested, tryptophan was used at concentrations of 0 (○), 0.005 M (▪), 0.01 M (▴) and 0.02 M (▾). RFU  =  relative fluorescence unit.*D*–*F*, Stern-Volmer plots (indicate fluorescence quenching (F_0_/F) as a function of quencher concentration [39]) for BODIPY with increasing concentrations of KI, tryptophan and acrylamide, respectively. F_0_ (fluorescence in the absence of quencher) and F (fluorescence in the presence of quencher).

LDs are often visualized by microscopy after staining with Nile red, osmium or BODIPY [35]–[37]. BODIPY has a lipophilic nature and exhibits high specificity for lipid droplets [26]; these qualities render the compound a good candidate to use as a probe for LDs in a fluorescent liquid high-throughput experiment. Based on fluorescence-quenched assays [38], we selected a hydrophilic quencher that would permit BODIPY to fluoresce only when localized inside LDs and not when present in solution (Figure 1). Three classical fluorescence quenchers (KI, acrylamide and tryptophan) were titrated against a 5 µM BODIPY solution. The emission spectra (485 nm excitation) showed that only KI quenched the fluorescence efficiently (Figure 2A–C). From Stern-Volmer plots, where the quenching efficiency is related to the total quencher concentration [39] it can be observed that quenching increased linearly with increasing concentrations of KI and tryptophane (Figure 2D,E) but not acrylamide (Figure 2F). From these plots, a Stern-Volmer constant of 44 and 14.1 was calculated for KI and acrylamide, respectively. KI was selected as the quencher because a higher F_0_/F ratio was reached with this compound as compared to tryptophan in the conditions tested.

**Figure 3:**
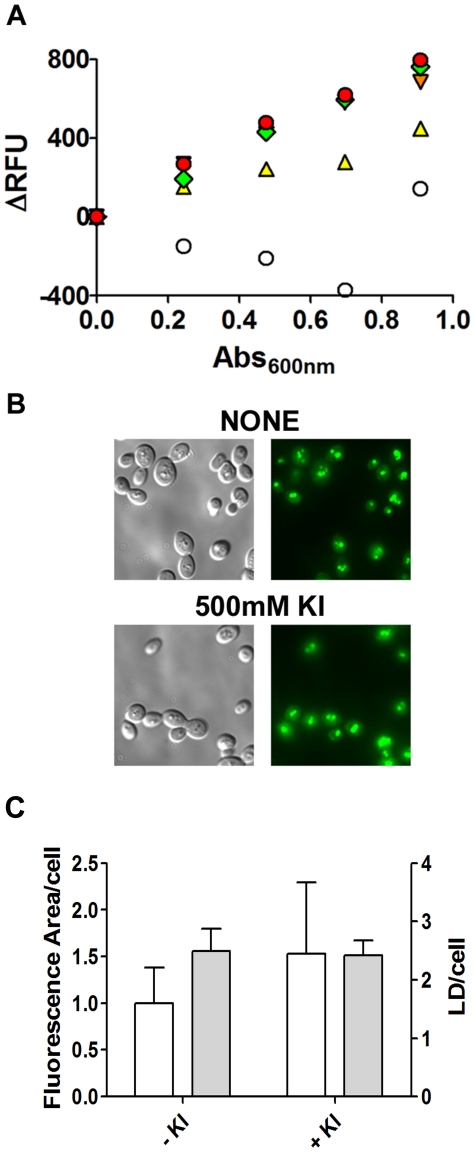
The BODIPY fluorescence signal quenched by iodide is recovered by the addition of cells to the medium. *A.* Increasing amounts of formaldehyde-fixed cells were added (subsequent 5 µl additions) to a 96-well plate containing 200 µl of 5 µM BODIPY in the presence of none (○), 0.1 M (▴, yellow), 0.25 M (▾, orange), 0.50 M (♦, green) or 1 M (•, red) KI. Fluorescence intensity (ex/em = 485/510 nm) and absorbance (600 nm) were recorded. A representative experiment is shown. *B.* Cells grown in YPD to stationary phase were formaldehyde-fixed, washed and resuspended to 10 DO 600 nm/ml. An aliquot of 5 µl of cells was mixed with 5 µl BODIPY solution (10 µM) in the absence (upper panel) or presence of 500 mM KI (lower panel). Stained cells were analyzed by fluorescence microscopy (100x magnification). *C*. Microscopy images were analyzed. The total fluorescence area/cell was determined and expressed in pixels/cell (white bars). LDs per cell were quantified using the same images (gray bars). Data are for at least 50 individual cells. No statistical difference was found between data collected from images stained with 5 µM Bodipy with or without 500 mM KI.

When fixed-formaldehyde cells were added to a BODIPY-KI-quenched solution, fluorescence was recovered (Figure 3A). The best linearity between the number of cells and recovered fluorescence was obtained using KI in the quenching medium at a concentration ranging from 0.25 M to 1.0 M. For subsequent experiments, the reading solution contained 500 mM KI and 5 µM BODIPY. To discard artifacts, we verified by microscopy that KI did not quench the fluorescence of LDs stained with BODIPY. The fluorescence area/cell and the number of LDs did not change significantly when 500 mM KI was added, suggesting that iodide is excluded from LDs (Figure 3B and C). This phenomenon allows researchers to measure the levels of lipid droplets within the cells in an easy and inexpensive manner (Figure 1). Some fluorophores exhibit a red*-*shift of the fluorescence spectra when in a non-polar environment, but the absorbance and emission spectra of BODIPY were unaltered, showing maximum absorbance at 485 nm and maximum emission at 510 nm with excitation at 485 nm (Figure S3).

### Sensitivity of the LFR assay

**Figure 4:**
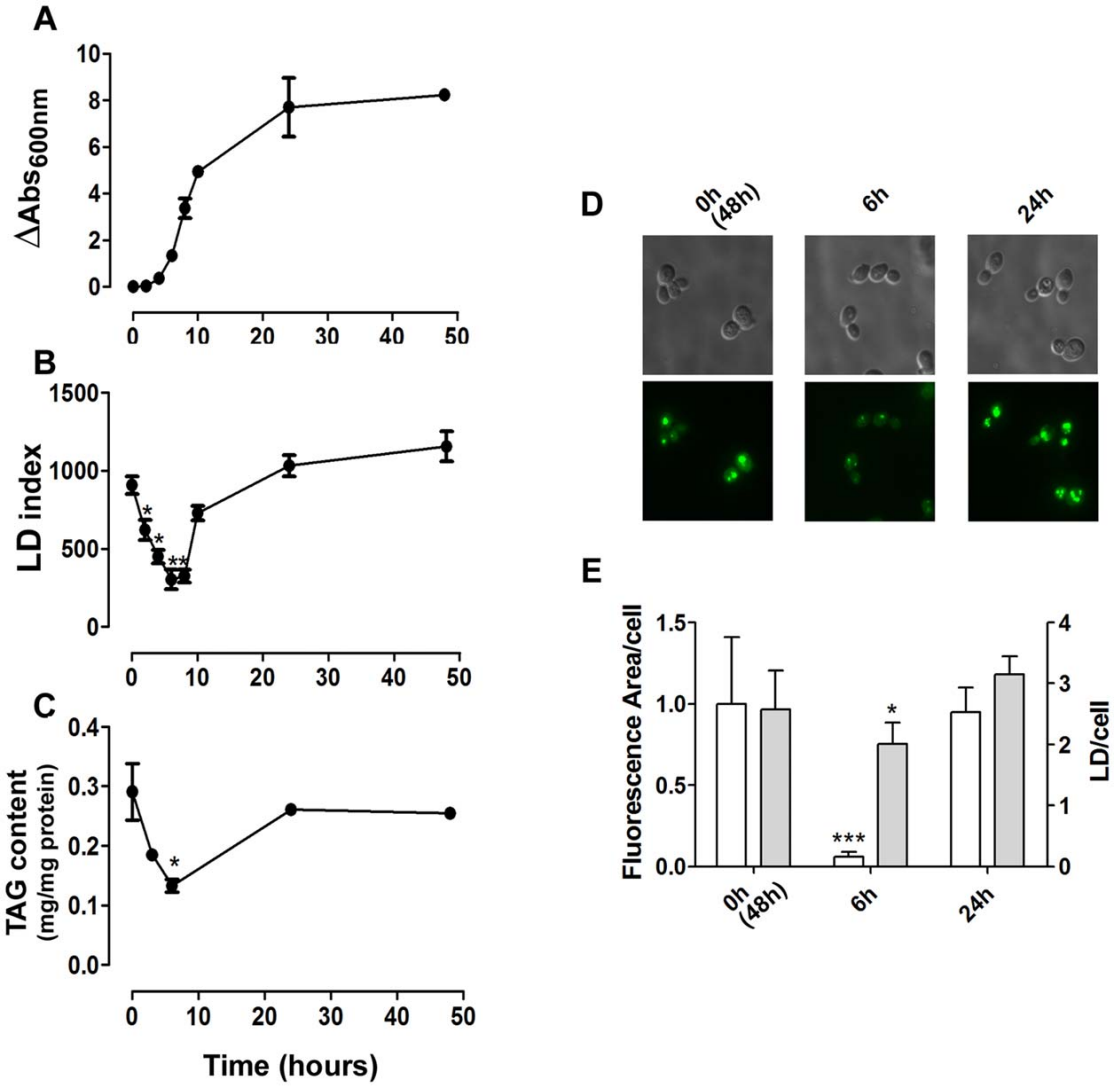
The liquid fluorescence recovery (LFR) assay can detect LD dynamics. *A.* Stationary yeast cells pre-grown for 48 hours, were inoculated into fresh YPD medium and cellular growth was recorded by measuring absorbance at 600 nm over the course of 48 hours (n = 3± S.D.). *B.* Aliquots of cells were withdrawn during growth and fixed in formaldehyde. The LD index was determined (fluorescence/OD cells) using the LFR assay (n = 3± S.D.) *C.* The triacylglycerol content was measured during growth. *D and E*. LD content was determined by fluorescence microscopy. Cells were grown for 0 (corresponding to 48 h pre-grown cells), 6 and 24 hours in YPD, incubated with BODIPY and photographed by fluorescence microscopy. The total fluorescence area/cell (white bars) were determined and expressed in pixels/cell (white bars). LDs per cell were quantified using the same images (gray bars). Data are for at least 50 individual cells. *p<0.05, ** p<0.01, ***p<0.001, in comparison to WT values.

To assess whether our method was able to detect small fluctuations in LD levels, we quantified this parameter during yeast growth as LDs are actively metabolized during growth [23], [40] (Figure 4A, B). Results obtained with the LFR assay (Figure 4B) were similar to those obtained by quantifying the TAG content (Figure 4C) or through microscopic analysis, from which we calculated the total fluorescence area/cell. Furthermore it did not reflect the number of LDs/cell that does not vary as much as the size of LDs along the growth curve (Figure 4D, E). The results were also in agreement with previous results obtained by microscopic analysis [23] and biochemical quantification of cellular TAG [40], which indicated that yeast cells consume LDs when diluted in fresh medium. After six hours, LDs are re-synthesized reaching highest levels at stationary phase [23], [40]. This result demonstrates that our assay was able to detect small physiological lipid stores fluctuations during yeast growth without the need for time-consuming image analysis.

### High-throughput experiment employing the LFR assay

**Figure 5:**
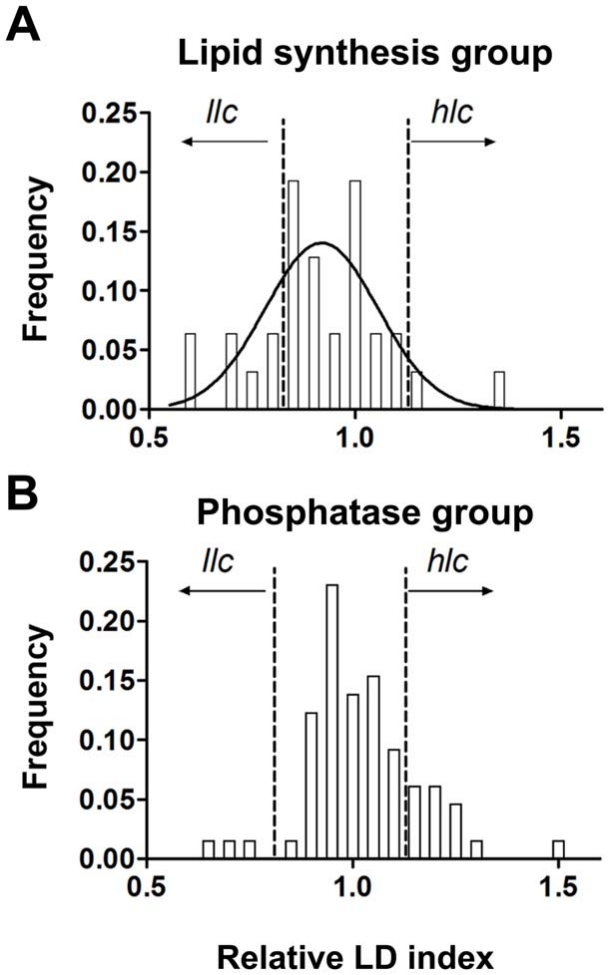
Statistical analysis of a high-throughput experiment employing the LFR assay. LDs were quantified by the LFR assay. The frequency distribution of the relative LD index of strains from the lipid synthesis group (A, n = 31) and protein phosphatase group (B, n = 65) was calculated. Data were collected from four independent experiments as described in the Materials and Methods section. The D'Agostino & Pearson normality test was employed at P<0.05. The lipid synthesis group was normally distributed (p = 0.281), while the protein phosphatase group failed to pass the normality test (p = 0.016). The arbitrary cohort (±20% in relation to WT values) is indicated in the graphs by a dashed line (*llc*, low lipid droplet content and *hlc*, high lipid droplet content).

We employed the LFR assay to study the involvement of protein phosphatases in LD metabolism. In this high-throughput experiment, we screened 96 null strains as well as the BY4741 WT strain (14 independent WT clones). We subdivided the strains in two groups. The first was a reference group, referred to as the lipid synthesis group. This reference group included 31 null strains for genes encoding LD-associated enzymes and/or enzymes involved in the metabolism of sterols, the metabolism of triglycerides and the biosynthesis of glycerolipids (Table 1) [41]. The second group, referred to as the phosphatase group, was composed of 65 null strains with all known non-essential protein phosphatases and non-essential phosphatase regulatory subunits (Table 2), according to the annotation in the Yeast Genome Database (www.yeastgenome.org, October 2009). As LD content varies substantially during growth (see Figure 4B) and not all strains have the same growth rate, we decided to asses LD content in strains grown to stationary phase, which was attained for all strains after 48 hours of seeding. A histogram of the distribution of the lipid indexes for all strains was analyzed and an index for cataloguing strains (as containing a higher LD index (*hlc*) or a lower LD index (*llc*)) was elaborated using an arbitrary cohort of ±20% in relation to the LD index of WT strains (Figure 5). When the mean LD index of the lipid synthesis group was compared to that of the WT strain, no significant difference was observed (means were 0.9539 and 0.9135, respectively p = 0.276). The same trend was not observed for the mean LD index of the protein phosphatase group, which was significantly higher than the WT sample (1.023, p = 0.0164) and included an over-representation of *hlc* strains. Interestingly, this result indicates that lipid accumulation is more likely provoked by the random loss of protein phosphatase function as opposed to the random loss of function among proteins involved in lipid metabolism. After analyzing the groups as a whole, individual strains were catalogued as having higher (*hlc*) or lower LD content (*llc*) (Tables 1 and 2).

**Table 1:** Lipid synthesis null strains screened by LFR assay.

NAME	SYSTEMATIC	BIOLOGICAL PROCESS	MEAN	S.D.	P
HMG2§	***YLR450W***	***STE synthesis***	**0.58**	**0.19**	**<0.001**
DGA1§,*	***YOR245C***	***TAG synthesis***	**0.59**	**0.16**	**<0.001**
ERG4§,*	***YGL012W***	***STE synthesis***	**0.70**	**0.18**	**0.003**
ERG5§,*	***YMR015C***	***STE synthesis***	**0.71**	**0.28**	**0.007**
ARE2§	***YNR019W***	***SE synthesis***	**0.76**	**0.21**	**0.023**
LRO1	*YNR008W*	*TAG synthesis*	0.81	0.09	0.069
DPP1	*YDR284C*	*TAG synthesis*	0.81	0.19	0.095
LPP1	*YDR503C*	*TAG synthesis*	0.84	0.12	0.152
ERG2	*YMR202W*	*STE synthesis*	0.84	0.06	0.164
GPT2	*YKR067W*	*TAG synthesis*	0.85	0.06	0.178
CSG2	*YBR036C*	*Ceramide synthesis*	0.85	0.26	0.233
TGL5	*YOR081C*	*TAG lipase*	0.86	0.17	0.265
AYR1	*YIL124W*	*TAG synthesis*	0.87	0.19	0.317
LAC1	*YKL008C*	*Ceramide synthesis*	0.88	0.09	0.359
HMG1	*YML075C*	*STE synthesis*	0.88	0.15	0.380
SUR2	*YDR297W*	*Ceramide synthesis*	0.91	0.01	0.048
ARE1	*YCR048W*	*SE synthesis*	0.91	0.10	0.626
IPT1	*YDR072C*	*Ceramide synthesis*	0.93	0.09	0.782
LAG1	*YHL003C*	*Ceramide synthesis*	0.94	0.01	0.577
TGL1	*YKL140W*	*SE hydrolase*	0.99	0.03	0.189
YEH2	*YLR020C*	*SE hydrolase*	1.00	0.08	0.540
ERG24	*YNL280C*	*STE synthesis*	1.00	0.14	0.546
SCS7	*YMR272C*	*Ceramide synthesis*	1.00	0.12	0.531
ERG6	*YML008C*	*STE synthesis*	1.01	0.20	0.507
SCT1	*YBL011W*	*TAG synthesis*	1.01	0.10	0.450
SUR1	*YPL057C*	*Ceramide synthesis*	1.03	0.04	0.332
YEH1	*YLL012W*	*SE hydrolase*	1.05	0.03	0.002
TGL4	*YKR089C*	*TAG lipase and SE hydrolase*	1.09	0.14	0.095
ERG3	*YLR056W*	*STE synthesis*	1.10	0.27	0.107
SLC1¥	***YDL052C***	***TAG synthesis***	**1.17**	**0.05**	**0.022**
TGL3¥,*	***YMR313C***	***TAG lipase***	**1.37**	**0.17**	**<0.001**

*7 out of 31 strains showed abnormal LP levels. Statistical analysis was performed against WT values (mean fluorescence  = 0.95, S.D.  = 0.15, n = 14). It was defined a cutoff using an arbitrary 20% difference between the mutant and WT relative fluorescence means. Phenotypes referred as llc*

*(§low lipid content,)*

*and hlc*

*(¥high lipid content,)*

*were marked in bold. Gene descriptions based in SGD (* www.yeastgenome.org *).*

*strains identified in a previously published screening for LP [*25*] *.*

**Table 2:** Phosphatase null strains screened by LFR assay.

NAME	SYSTEMATIC	DESCRIPTION	MEAN	S.D.	P
SIT4§	***YDL047W***	***Type 2A-related protein phosphatase***	**0.63**	**0.09**	**<0.001**
REG1§	***YDR028C***	***Regulatory subunit of type 1 protein phosphatase Glc7p***	**0.68**	**0.16**	**0.002**
SAP190§	***YKR028W***	***Regulatory subunit of Sit4 phosphatase***	**0.73**	**0.07**	**0.007**
SIS2	*YKR072C*	*Regulatory subunit of type 1 protein phosphatase Ppz1*	0.86	0.07	0.223
PIG2	*YIL045W*	*Subunit for the type-1 protein phosphatase Glc7p*	0.89	0.12	0.450
LTP1	*YPR073C*	*Protein tyrosine phosphatase*	0.90	0.12	0.518
RTS1	*YOR014W*	*Regulatory subunit of protein phosphatase 2A*	0.91	0.07	0.598
YBR051W		*Dubious ORF, partially overlaps REG2*	0.92	0.29	0.809
PTC6	*YCR079W*	*Type 2C protein phosphatase*	0.92	0.10	0.636
GIP2	*YER054C*	*Subunit for the type-1 protein phosphatase Glc7p*	0.92	0.11	0.646
YVH1	*YIR026C*	*Phosphotyrosine-specific protein phosphatase*	0.92	0.13	0.653
CNA1	*YLR433C*	*Subunit of calcineurin, a type 2B protein phosphatase*	0.92	0.10	0.661
PTC3	*YBL056W*	*Type 2C protein phosphatase*	0.93	0.17	0.725
PTC5	*YOR090C*	*Type 2C protein phosphatase*	0.93	0.08	0.722
GLC8	*YMR311C*	*Subunit for the type-1 protein phosphatase Glc7p*	0.93	0.04	0.457
TIP41	*YPR040W*	*Interacts physically and genetically with Tap42p, regulates Sit4*	0.93	0.09	0.781
SIW14	*YNL032W*	*Phosphotyrosine-specific protein phosphatase*	0.94	0.10	0.821
CNB1	*YKL190W*	*Subunit of calcineurin, a type 2B protein phosphatase*	0.94	0.08	0.897
RTS3	*YGR161C*	*Regulatory subunit of protein phosphatase 2A*	0.95	0.10	0.930
PSR2	*YLR019W*	*Protein phosphatase involved in the general stress response*	0.95	0.04	0.992
OCA2	*YNL056W*	*Putative protein tyrosine phosphatase*	0.95	0.22	0.997
SDP1	*YIL113W*	*MAP kinase phosphatase*	0.96	0.17	0.971
YNL010W		*Unknown function; similar to phosphoserine phosphatases*	0.96	0.08	0.948
PTP1	*YDL230W*	*Phosphotyrosine-specific protein phosphatase*	0.96	0.09	0.932
SIP5	*YMR140W*	*Unknown function; interacts with both the Reg1p/Glc7p*	0.96	0.14	0.928
CMP2	*YML057W*	*Subunit of calcineurin, a type 2B protein phosphatase*	0.97	0.12	0.866
YGR203W		*Phosphatase with sequence similarity to Cdc25p, Arr2p and Mih1p*	0.97	0.14	0.814
PPH22	*YDL188C*	*Type 2A protein phosphatase*	0.99	0.14	0.636
PPT1	*YGR123C*	*Ser/thr phosphatase with similarity to human phosphatase PP5*	0.99	0.14	0.622
PPG1	*YNR032W*	*Putative serine/threonine protein phosphatase*	1.00	0.07	0.596
PTC2	*YER089C*	*Type 2C protein phosphatase*	1.01	0.15	0.522
RRD2	*YPL152W*	*Activator of the phosphotyrosyl phosphatase activity of PP2A*	1.01	0.09	0.474
MIH1	*YMR036C*	*Protein tyrosine phosphatase*	1.01	0.07	0.458
PIG1	*YLR273C*	*Subunit for the type-1 protein phosphatase Glc7p*	1.01	0.15	0.467
GIP1	*YBR045C*	*Subunit for the type-1 protein phosphatase Glc7p*	1.02	0.04	0.048
PHO13	*YDL236W*	*Alkaline phosphatase, also has protein phosphatase activity*	1.02	0.09	0.382
SAP4	*YGL229C*	*Regulatory subunit of Sit4 phosphatase*	1.03	0.16	0.376
NBP2	*YDR162C*	*Recruitment of phosphatase Ptc1p to the Pbs2p-Hog1p complex*	1.04	0.07	0.264
PTP3	*YER075C*	*Phosphotyrosine-specific protein phosphatase*	1.05	0.17	0.267
YER121W		*Putative protein of unknown function*	1.05	0.07	0.249
VHS3	*YOR054C*	*Regulatory subunit of type 1 protein phosphatase Ppz1*	1.05	0.15	0.255
NEM1	*YHR004C*	*Probable catalytic subunit of Nem1p-Spo7p phosphatase*	1.05	0.10	0.246
PPZ1	*YML016C*	*Type 1 protein phosphatase*	1.05	0.16	0.221
PSR1	*YLL010C*	*Protein phosphatase involved in the general stress response*	1.05	0.11	0.210
PSY4	*YBL046W*	*Regulatory subunit of type 2A protein phosphatase Pph3*	1.06	0.19	0.200
SAP155	*YFR040W*	*Regulatory subunit of Sit4 phosphatase*	1.06	0.14	0.179
PPQ1	*YPL179W*	*Putative protein serine/threonine phosphatase*	1.08	0.21	0.147
PPH3	*YDR075W*	*Type 2A protein phosphatase*	1.09	0.04	0.091
PSY2	*YNL201C*	*Regulatory subunit of type 2A protein phosphatase Pph3*	1.09	0.13	0.098
REG2	*YBR050C*	*Regulatory subunit of type 1 protein phosphatase Glc7p*	1.09	0.05	0.086
PTP2	*YOR208W*	*Phosphotyrosine-specific protein phosphatase*	1.12	0.22	0.057
OCA1	*YNL099C*	*Putative protein tyrosine phosphatase*	1.12	0.25	0.058
PAM1	*YDR251W*	*Essential protein of unknown function*	1.13	0.15	0.028
PTC7¥	***YHR076W***	***Type 2C protein phosphatase***	**1.14**	**0.22**	**0.028**
PPS1¥	***YBR276C***	***Protein phosphatase with specificity for Ser, Tthr and Tyr residues***	**1.15**	**0.10**	**0.017**
SAP185¥	***YJL098W***	***Regulatory subunit of Sit4 phosphatase***	**1.16**	**0.18**	**0.015**
PPZ2¥	***YDR436W***	***Type 1 protein phosphatase***	**1.18**	**0.12**	**0.007**
MSG5¥	***YNL053W***	***Dual-specificity protein phosphatase***	**1.18**	**0.21**	**0.009**
PTC4¥	***YBR125C***	***Type 2C protein phosphatase***	**1.20**	**0.19**	**0.004**
SPO7	*YAL009W*	*Regulatory subunit of Nem1p-Spo7p phosphatase*	1.22	0.30	0.172
PPH21¥	***YDL134C***	***Type 2A protein phosphatase***	**1.23**	**0.17**	**0.002**
GAC1¥	***YOR178C***	***Subunit for the type-1 protein phosphatase Glc7p***	**1.25**	**0.14**	**0.001**
BNI4¥	***YNL233W***	***Subunit for the type-1 protein phosphatase Glc7p***	**1.26**	**0.11**	**0.002**
PTC1¥	***YDL006W***	***Type 2C protein phosphatase***	**1.28**	**0.26**	**<0.001**
TPD3	*YAL016W*	*Regulatory subunit of protein phosphatase 2A*	1.52	0.39	0.127

*13 out of 65 strains showed abnormal LP levels. Statistical analysis was performed against WT values (mean fluorescence  = 0.95, S.D.  = 0.15, from 14 WT samples). It was defined a cutoff using an arbitrary 20% difference between the mutant and WT relative fluorescence means. Phenotypes referred as llc*

*(§low lipid content,)*

*and hlc*

*(¥high lipid content,)*

were marked in bold. Gene descriptions based in SGD (www.yeastgenome.org).

Most of the results obtained in the lipid synthesis group corroborated results previously reported in the literature. Five strains were identified as *llc* strains (*hmg2*, *dga1*, *erg4*, *erg5*, *are2*) and two were identified as *hlc* strains (*slc1* and *tgl3*). Interestingly, among the *llc* strains we identified were *dga1*, which encodes an enzyme responsible for the last step in TAG synthesis (acyl-CoA-dependent pathway); *hmg2*, which encodes HMG-CoA reductase, which catalyzes the rate-limiting step in ergosterol synthesis; and *are2*, which esterifies sterols. Deletion of Dga1p and Are2p would lead to diminished levels of TAG and sterol/steryl esters levels [42], respectively. In the *hlc* group, we identified *tgl3* which encodes a TAG lipase. Deletion of this enzyme increases TAG levels [12]. In the same group, we also identified *slc1*, which encodes a 1-acylglycerol-3-phosphate acyltransferase involved in phosphatidic acid biosynthesis. The exact role of this protein in LD formation has not been demonstrated, but its deletion led to higher TAG levels in cells [43]. Reinforcing our results, in a previously published screening, *dga1* and *tgl3* were identified as having diminished and increased number of LDs, respectively [44]. Although we classified the *erg4* and *erg5* mutants as *llc* strains, these strains have been reported to have more LDs than WT strains when analyzed by fluorescent microscopy [44]. Microscopic analysis confirmed that these strains have more LDs than WT strains but show diminished total fluorescence area (Figure S4), thus indicating a reduction in LD size. These results are interesting because they suggest that fluctuations measured by LFR assay are due to increased total fluorescence area. However the above results do not show if differences assayed in the LFR assay are due to different levels of SE or TAG, or both. To test this we have compared the fluorescence lipid droplet index and fluorescence area/cell of *are1are2* and *dga1lro1* cells, lacking SE and TAG, respectively (Figure S5). The mean fluorescence area/cell was 40% lower in *are1are2* compared to WT while the LFR assay showed an 8% reduction. The *dga1lro1* presented a diffuse pattern without distinguishable LDs when stained with BODIPY using the same exposure times as WT being not valid to compare the fluorescence area/cell, however with longer exposure times it was possible to visualize a few LDs/cell. The LFR assay detected a 40% reduction in *dga1lro1* LD index. These results show that the LFR assay detects the total area/cell of lipid droplets composed of mainly TAG or SE and that it is more sensitive than fluorescence microscopy studies.

#### The Ser/Thr protein phosphatase Sit4p regulates Snf1 kinase phosphorylation in association with its regulatory subunit Sap190p

**Figure 6:**
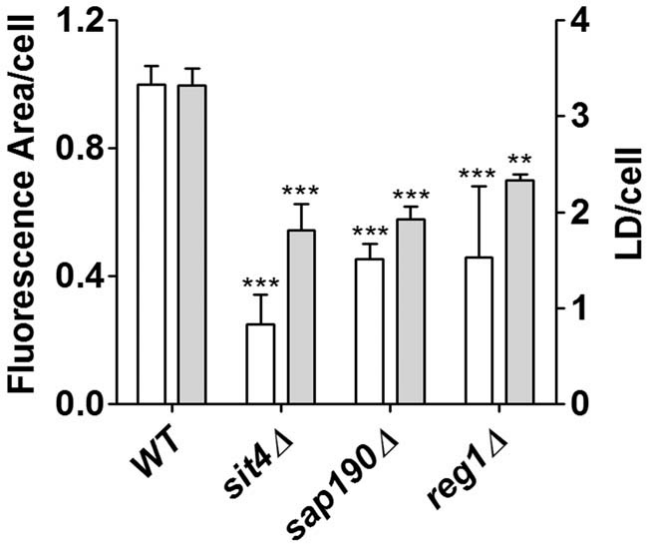
LDs size and content in *llc* phosphatase mutants. The total area/cell and the number of LDs were analyzed from microscopic images of *WT*, *sit4*, *sap190*, *reg1* grown in YPD medium for 48 hours, as described. ** p<0.01, ***p<0.001, in comparison to WT values.

Among the protein phosphatase group, three strains were marked as llc (sit4, sap190 and reg1). Two of these genes are related: *SIT4* encodes the catalytic subunit of a type 2A-like Ser-Thr phosphatase and *SAP190* encodes one of its regulatory subunits. *REG1* codes for a regulatory subunit of Glc7 phosphatase, an essential type-1 protein phosphatase. On the other hand, 10 strains were marked as hlc. Three of these were type 2C protein phosphatases (*ptc1*, *ptc4*, *ptc7*); three were related to type 1 protein phosphatases, a catalytic subunit (*ppz2*) and Glc7 regulatory subunits (*gac1* and *bni4*). Pph21p is a type 2A protein phosphatase and Sap185p is a regulatory subunit of Sit4p. Other null strains marked as *hlc* were *pps1* and *msg5*. These results call for a new perspective on the topic at hand and indicate that an intricate regulation of protein phosphorylation is involved in the regulation of LD metabolism by protein phosphatases. Since previous screens have missed *sit4*, *sap190* and *reg1* as having less LDs we counted LDs and measured the fluorescent area/cell (Figure 6). Results show that both parameters are significantly lower compared to WT.

We further studied *llc* hits. Together with Tap42p, Sit4p is known for its participation in the TOR pathway, which senses nutrient availability and coordinates cell growth [45]. Sit4p was first identified as a type2A-related phosphatase activated by extracellular ceramide [46]. Sit4p is also regulated by four other subunits thought to act as targeting subunits: Sap4p, Sap155p, Sap185p and Sap190p. These proteins have different or overlapping roles in the regulation of the cell cycle [47] and in translation control [48]. Reg1p directs Glc7p to its targets, participating in the regulation of several processes such as glucose de-repression and glycogen synthesis [49], [50]. Reg1p plays a role in energetic metabolism by interacting with Snf1 kinase, a Ser/Thr kinase homologue to mammalian AMPK responsible for maintaining energy homeostasis. Upon depletion of glucose in the medium, Snf1 is phosphorylated by upstream kinases (called Snf1-activating kinases, SAKs) and becomes active [51]. Its dephosphorylation is promoted by Glc7p phosphatase and is dependent on Reg1p, as the *reg1* strain displays hyperphosphorylated Snf1p [52]. However, this finding must be investigated further because greater Reg1p-Snf1p interaction is observed in low-glucose conditions when Snf1p is more active [53]. Snf1p kinase, in turn, phosphorylates a key enzyme in FA biosynthesis, acetyl-CoA carboxylase (Acc1), which inhibits the activity of Acc1. It has been shown that deletion of *SNF1* increases Acc1 activity as *snf1* strain is more resistant to soraphen A which is a potent inhibitor of acetyl-CoA carboxylase – the more resistant a strain is, the greater is its level of acetyl-CoA carboxylase activity [24], [25], [54].

**Figure 7:**
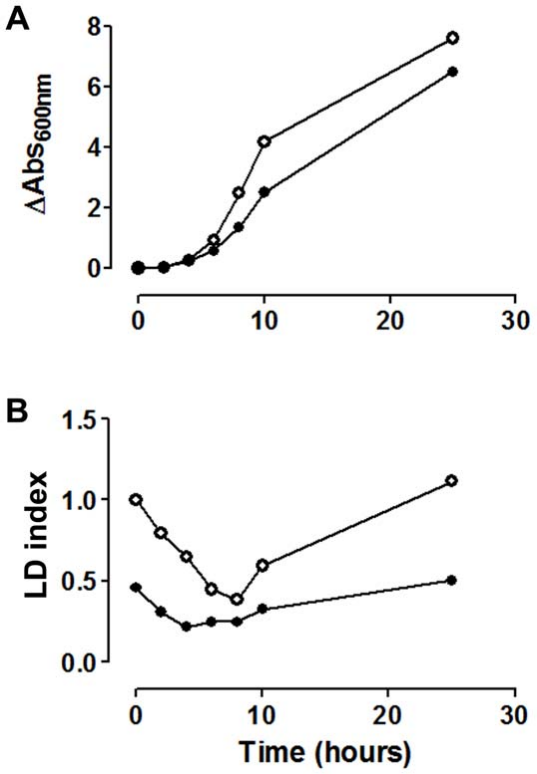
LD dynamics is impaired in the *sit4* strain. *A.* Stationary yeast cells (WT, ○, and *sit4Δ*, •) grown for 48 in YPD were inoculated into fresh medium. Cellular growth was recorded by measuring absorbance at 600 nm over the course of 25 hours. *B.* Lipid droplet levels were determined by LFR assay during growth as described in Figure 4. LD index was normalized by stationary WT value. The mean of two independent experiments is shown.

**Figure 8:**
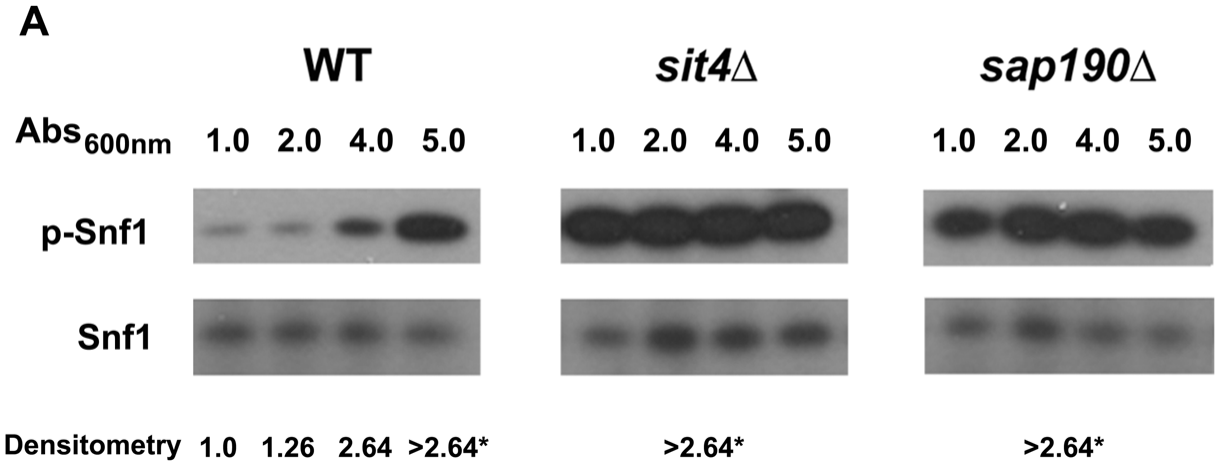
Snf1 kinase phosphorylation levels during growth. WT, *sit4* and *sap190* cells were grown in YPD at 30°C while shaking. Aliquots of the culture were harvested at different times during growth (OD 600 nm of 1, 2, 4 or 5), and protein was extracted using the rapid NaOH extraction method [31]. SDS-PAGE gels were loaded with 0.1 OD _600 nm_ cells per well. Snf1 phosphorylation was analyzed by western blot using anti-phospho AMPKα antibody (P-SNF1, upper panel). As a control for loading, total Snf1 was revealed with anti-SNF1 antibody (lower panel). For autoradiography the films were exposed for the minimum time necessary to detect the phosphorylation signal for WT grown to OD 1.0 which was used as reference. The numbers below the figure represent the densitometry analysis of the P-SNF1/SNF1 ratio (n = 3). Signals were linear unless marked by*.

**Figure 9:**
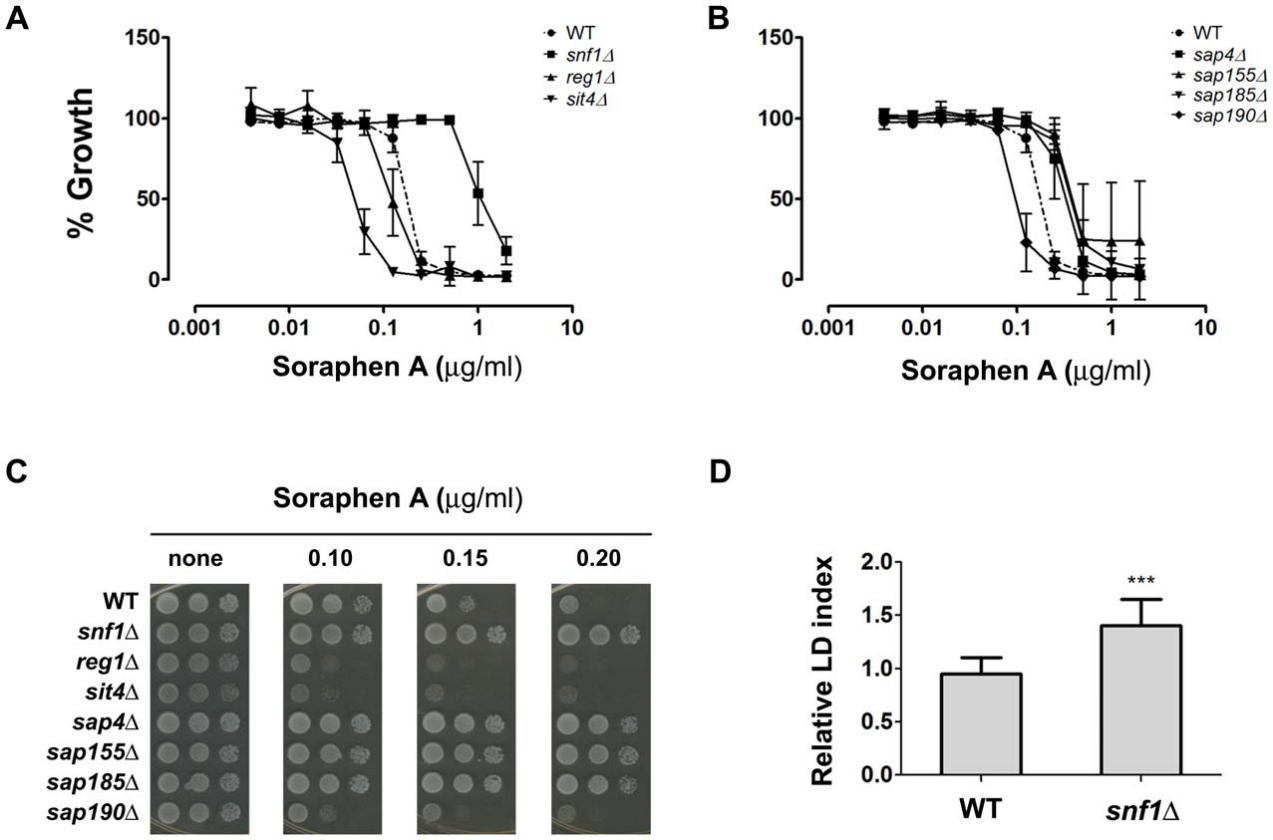
Soraphen A resistance profiles. Soraphen A resistance was determined by the microtiter assay and the spot assay. *A and B.* Serial dilutions of soraphen A were used for the indicated strains (n = 3± S.D.) IC_50_ were estimated (wt  = 0.19, *snf1  = 0.95*, *reg1*  = 0.11, *sit4*  = 0.05, *sap4*  = 0.36, *sap155*  = 0.38, *sap185*  = 0.38 and *sap190* = 0.1 µg/ml). *C.* For the spot assay, serial dilutions (10^7^, 10^6^ and 10^5^ cells/mL, from left to right) of a cellular suspension were seeded onto plates containing YPD solid medium in the presence of increasing concentrations of soraphen A and incubated at 30°C for two days. *D.* Relative LD index of *snf1* cells from a stationary culture in YPD medium was determined by LFR assay, as described. (n = 3,± S.D.) (***p<0.001).

We further investigated whether diminished LD content in *sit4* strain was related to a lower rate of synthesis by following its metabolism in a growing culture (Figure 7). Results show that *sit4* strain was able to consume LDs after inoculating in fresh medium, during the lag phase of growth, after which the rate of synthesis of LDs was low compared to WT. This result points to a deficient regulation of acetyl-CoA carboxylase activity. In fact, when the phosphorylation state of Snf1p was analyzed during growth, we observed that Snf1p is constitutively phosphorylated in *sit4* strain, while it is dephosphorylated in the WT strain during the early log phase. Furthermore, phosphorylation of Snf1p increases throughout culture growth and reaches a maximum during the late exponential phase, when the rate of lipid biosynthesis may have started to slow down (Figure 8). Interestingly Snf1p was also constitutively phosphorylated in *sap190* strain during all phases of culture growth. To further confirm that Snf1p is in its active state in *sit4* strain (thus inhibiting acetyl-CoA carboxylase activity), we screened the sensitivity of these null mutant strains to soraphen A (Figure 9). Results revealed that *sit4* strain is more sensitive to soraphen A. *reg1* strain has also been shown to be more sensitive to soraphen A, and it has been shown that in this strain, Snf1p is in its active state [55] and probably leads to an inhibition of acetyl-CoA carboxylase. The results observed were consistent with the classification of *sit4* and *reg1* as *llc* strains. As a control, we tested the sensitivity of the *snf1* strain. As expected, this resulted in greater resistance to soraphen A due to the activation of acetyl-CoA carboxylase activity [25], [34]. Furthermore, we observed that the *snf1* strain presented an LD index that was higher than that of the wild-type (Figure 9D).

**Figure 10:**
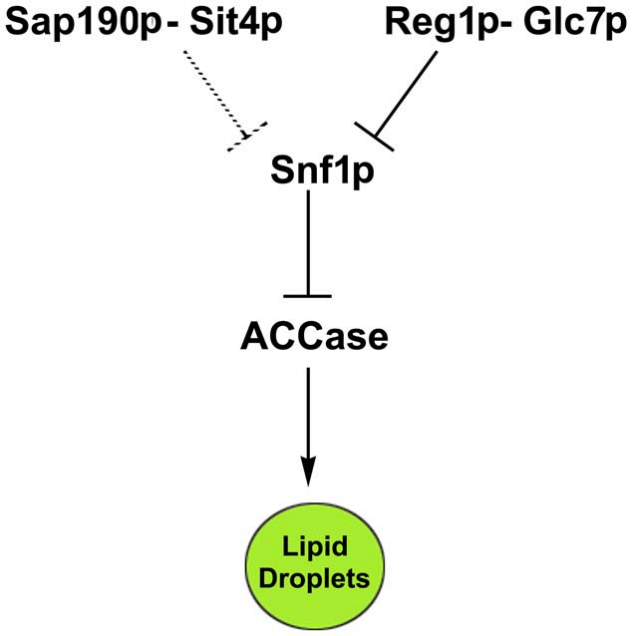
Sit4p orchestrates lipid metabolism in response to nutrients. Snf1 kinase, a central regulator of energetic homeostasis, when phosphorylated and active, inhibits acetyl CoA carboxylase activity impairing fatty acids synthesis diminishing LD levels. It is dephosphorylated by Reg1/Glc7 phosphatase as glucose levels in the medium increase [50]. Our results suggest that the Sap190-Sit4 pair also participates in Snf1 dephosphorylation and links the TOR pathway to the AMP/ATP-sensitive pathway, mediated by Snf1.

The results of testing the sensitivity of SAP proteins to soraphen A show that *sap190* strain leads to a probable inhibition of acetyl-CoA carboxylase activity, which reinforces its classification as an *llc* mutant. Interestingly, other Sap deletions led to an increase in soraphen A resistance, which is not correlated to an increase in lipid droplets. Such an effect can be explained because it is believed that Saps compete among themselves for Sit4p association and, thus, the deletion of one sap will increase the chances that Sit4 associates with the remaining Saps [47]. These results suggest that the Sit4p-Sap190p complex connects the TOR pathway to the SNF1/AMPK pathway to regulate lipid droplet metabolism (Figure 10).

## Discussion

There is increasing interest in LD metabolism in the field of biological science as more evidences connect its deregulation to several pathological processes, such as the inflammatory response [56], insulin resistance [57] and even neurodegenerative disorders [58], in addition to the known role of LD in lipid storage. However, there are still fundamental questions to be resolved regarding the signaling pathways involved in LD regulation. The methods employed in lipid studies have not facilitated great advances; such investigations still rely on time-consuming and expensive microscopic studies or on organic solvent extraction and subsequent analysis. Working with such techniques requires well-trained researchers because the steps involved can be tricky and laborious. We propose an alternative, sensitive method for the study of LD dynamics, which can be applied in high-throughput experiments to yield reliable results.

BODIPY 493/503 is a BODIPY fluorophore derivative that, due to its lipophilic nature, accumulates in LDs. It is often employed in microscopic studies of these organelles as an alternative to Nile red. In fact, Nile red acts more as a hydrophobic probe because it only fluoresces in hydrophobic environments in a manner that is proportional to the surrounding hydrophobicity; it has been shown that Nile red stains both membranes and LD [27], [28]. On the other hand, BODIPY is more specific than Nile red for LDs, as revealed by flow cytometry experiments [26], [29]. Furthermore, BODIPY seems to be less sensitive to environmental conditions, such as pH and polarity [59], which could be an advantage when comparing LD staining in different mutant strains. Once optimal conditions were determined, our assay was evaluated by following LD dynamics during culture growth. In this experiment (Figure 4) the size (total area/cell) and number of LDs was assayed by analyzing microscope images of cells stained with BODIPY and TAG levels were measured biochemically. Our fluorescence-based method provided results consistent with the TAG content and with the size of LDs. On the other hand, it did not correlate with the number of LDs which do not vary substantially along the growth curve, when compared to the size of LDs.

Analysis of a reference group comprising strains in which the genes for lipid synthesis and LD dynamics have been knocked out not only validated the entire screening but also reinforced the reliability of our method. In a previous screening in which the entire deletion library was analyzed microscopically to quantify LDs [44], at least two of our hits (*dga1* and *tgl3*) were identified as inducing abnormal phenotypes. However, *erg4* and *erg5* strains, which were identified as having increased LD number – phenotypes we confirmed microscopically – were identified in our screening as having lower fluorescence levels, which indicates lower lipid content. A plausible explanation is that, although they contain more LDs, these organelles are substantially smaller, which could, in turn, result in reduced BODIPY capture and thus less fluorescence. Interestingly, no relevant protein phosphatases were identified in the previously mentioned screening. In here, among 65 strains in which genes for protein phosphatases or related subunits were knocked out, 10 presented higher lipid droplet content while only 3 (*reg1*, *sit4* and its regulatory subunit *sap190*) presented lower lipid droplet content. This result suggests that a loss of protein phosphatase function is more likely to result in fluctuations in LDs size as compared to the number of LDs. By analyzing the overall results our attention was drawn to the involvement of three subunits of Glc7 phosphatase. *gac1* and *bni4* were identified as *hlc* while *reg1* as *llc*. Glc7p phosphatase interacts with different subunits each leading Glc7p to different substrates regulating several cellular processes as cell cycle, glycogen, sugar and lipid metabolism [60], [61]. Gac1p and Bni4p recruit Glc7p phosphatase to glycogen synthase Gsy2p [62] or the bud neck [63], respectively, and normally they may sequester some Glc7 phosphatase away from Snf1p. Their deletion might make more Glc7p available for Reg1p-Snf1p, thus increasing Snf1p dephosphorylation/inactivation, thereby increasing Acc1p activity and fatty acid synthesis. On the other hand interaction of Reg1p with Glc7p is necessary to dephosphorylate Snf1p. Its deletion leads to phosphorylation/activation of Snf1p, inhibiting the synthesis of LDs.

These three genes, *REG1*, *SIT4* and *SAP190*, have been identified in a previous screen as necessary for normal growth on oleic acid. The reason how the presence of Sap190p-Sit4p prevents oleate inhibition is not known [64]. Kohlwein et al [17] have shown that TAG synthesis plays an important role to buffer excess oleic acid, as strains lacking the acyltransferases Lro1p, Dga1p, Are1p, and Are2p are sensitive to this fatty acid. Together with our results, these observations suggest that *reg1*, *sit4* and *sap190* are necessary to induce synthesis of LDs in the presence of oleate, thus preventing its toxicity.

We further studied the role of Sit4p participation in lipid metabolism. The *sit4* strain LD dynamics showed a lower rate of LD synthesis after lag-phase, in comparison to WT (Figure 7). Although we cannot discard defects in LD synthesis or the degradation apparatus, the Sit4p deletion effect is likely due to the diminished availability of construction blocks such as FA. In fact, researchers believe that Sit4 phosphatase might dephosphorylate acetyl-CoA carboxylase, a key enzyme in fatty acid metabolism. However, experimental results have not been published [65]–[67]. Here, we showed that *sit4* deletion increases sensitivity to soraphen A, an acetyl-CoA carboxylase inhibitor. The same is observed for the deletion of *sap190*, a regulatory subunit of *SIT4*. Moreover, it is important to observe that *sap185* deletion induced a phenotype opposite to that elicited by *sap190* deletion; the *sap185* knockout strain was detected as a high lipid content strain during screening. Although SAPs compete for Sit4 binding [47] and have exhibited opposing functions [68], so far, no antagonistic relationship between Sap185p and Sap190p has been observed. More studies will be necessary to answer whether the opposing LD phenotypes resulting from these *SAP* genes deletions are related, indicating the existence of a novel antagonistic pair.

To explore a possible connection between TOR and Snf1 signaling pathways (because Snf1 takes part in nitrogen signaling and both are implicated in lifespan extension mechanisms), we investigated Snf1 phosphorylation status in the *sit4* strain. Surprisingly, the deletion of *sit4* (or its subunit *sap190*) leads to increased constitutive Snf1 phosphorylation, being more active. This finding would explain an increase in soraphen A sensitivity due to a decrease in acetyl-CoA carboxylase activity. Although we cannot discard the hypothesis that Sit4 directly dephosphorylates acetyl-CoA carboxylase, we provide evidence that Sit4 participation in lipid metabolism occurs through the regulation of Snf1 phosphorylation status and links the TOR pathway to the AMP/ATP-sensitive pathway, which is regulated by Snf1/AMPK.

## Supporting Information

Figure S1 Liquid fluorescence recovery assay (LFR assay). Schematic representation of the LFR assay, where cells are first fixed in 3.7% formaldehyde, washed and then added to BODIPY-quenched solution containing 5 µM BODIPY plus 500 mM KI. Fluorescence is recovered and detected with a fluorimeter (ex/em = 485/510 nm).
(1.46 MB TIF)

Figure S2 High-throughput screening of a knockout collection. Individual strains are pre-grown to stationary phase at 30°C and fixed in formaldehyde (3.7%). Cells are washed and added to a 96-well black-wall/clear-bottom plate. Fluorescence (ex/em = 485/510 nm) and absorbance (600 nm) are recorded. Three more subsequent readings were performed after the addition of cells to the wells in order to determine the LD index.
(0.46 MB TIF)

Figure S3 Fluorescence properties of BODIPY are not altered in the presence of cells. An aqueous solution of BODIPY (5 µM) was incubated in the presence of the indicated of none (Ο), 0.025 OD (square), 0.05 OD (Δ) and 0.1 OD (∇) of cells and the absorption spectra (upper panel) and emission (excitation at 485 nm) (lower panel) of BODIPY were recorded. Results are in agreement with information provided by the manufacturer (BODIPY Lipid Probes manual, available online at www.invitrogen.com), which states that BODIPY-derivative fluorescence parameters are insensitive to environmental conditions and not quenched by water.
(0.62 MB TIF)

Figure S4 LD content in *erg4* and *erg5* strains was determined by fluorescence microscopy. Cells were grown to stationary phase and incubated with BODIPY. The total fluorescence area/cell was determined and expressed in pixels/cell (white bars). LDs per cell were quantified using the same images (gray bars). Data provided are for at least 100 individual cells. LD index is indicated below the graph for each strain. *p<0.05, ** p<0.01, ***p<0.001, in comparison to WT values.
(1.59 MB TIF)

Figure S5 LD index correlates with either TAG or SE content. WT, *are1Δ are2Δ* and *dga1Δ lro1Δ* strains were grown to stationary phase and incubated with BODIPY. Images were captured for 17msec. A second capture (74msec) for *dga1Δ lro1Δ*, is also shown (last panel). At the bottom of the figure the LD index (LFR assay) (n = 3) and size (fluorescence area/cell LDs, microscopy images n = 100 cells) for each strain are shown. ** p<0.01, ***p<0.001, in comparison to WT values.
(2.78 MB TIF)
