# Supplementary figures and images for: A New Fluorescence-Based Method Identifies Protein Phosphatases Regulating Lipid Droplet Metabolism
Source: PLoS One. 2010 Oct 28;5(10):e13692. doi: 10.1371/journal.pone.0013692 (PMC2965658; doi:10.1371/journal.pone.0013692)

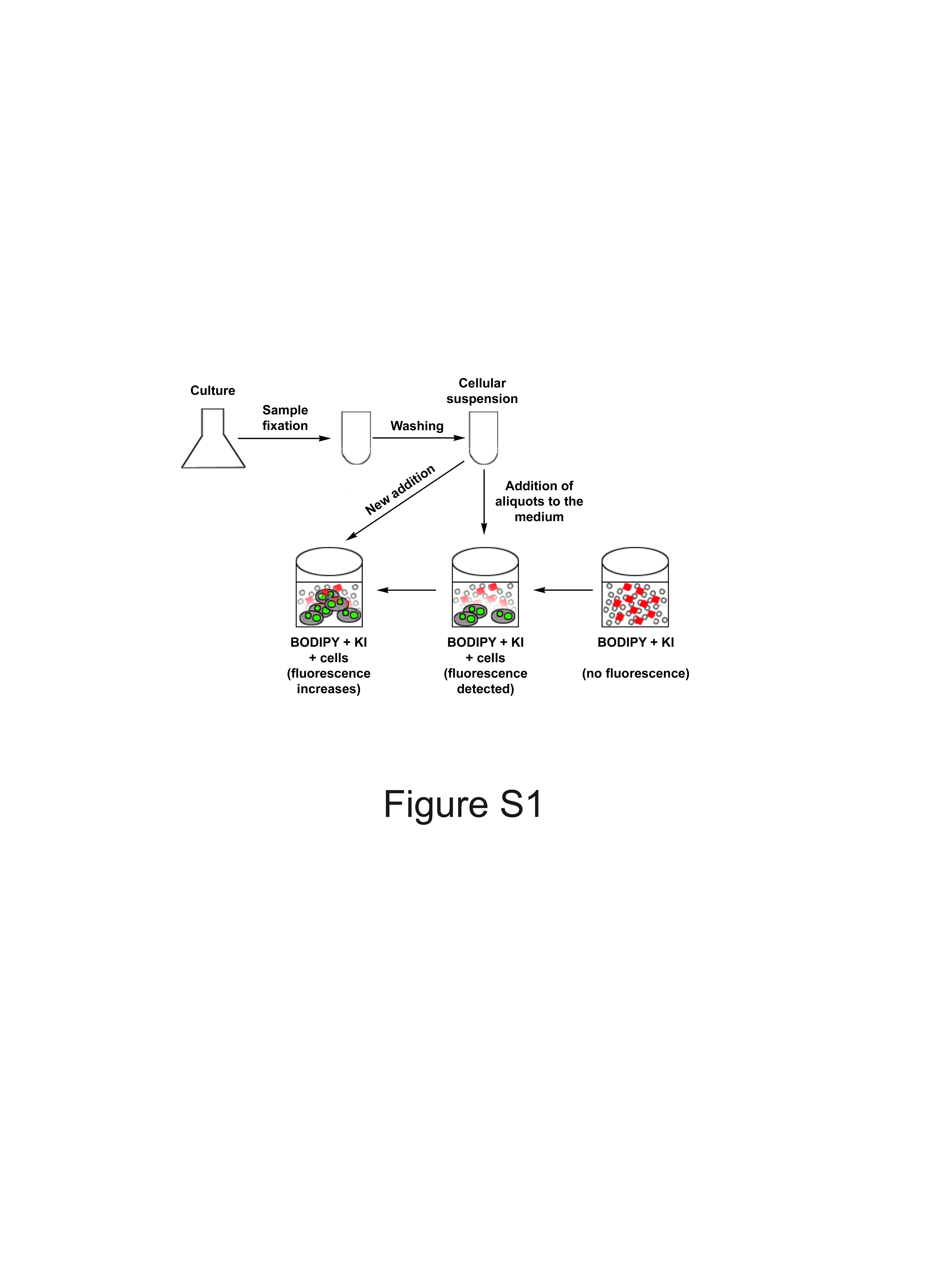

Supplement: Figure S1 — Liquid fluorescence recovery assay (LFR assay). Schematic representation of the LFR assay, where cells are first fixed in 3.7% formaldehyde, washed and then added to BODIPY-quenched solution containing 5 µM BODIPY plus 500 mM KI. Fluorescence is recovered and detected with a fluorimeter (ex/em = 485/510 nm). (1.46 MB TIF) [file pone.0013692.s001.tif]

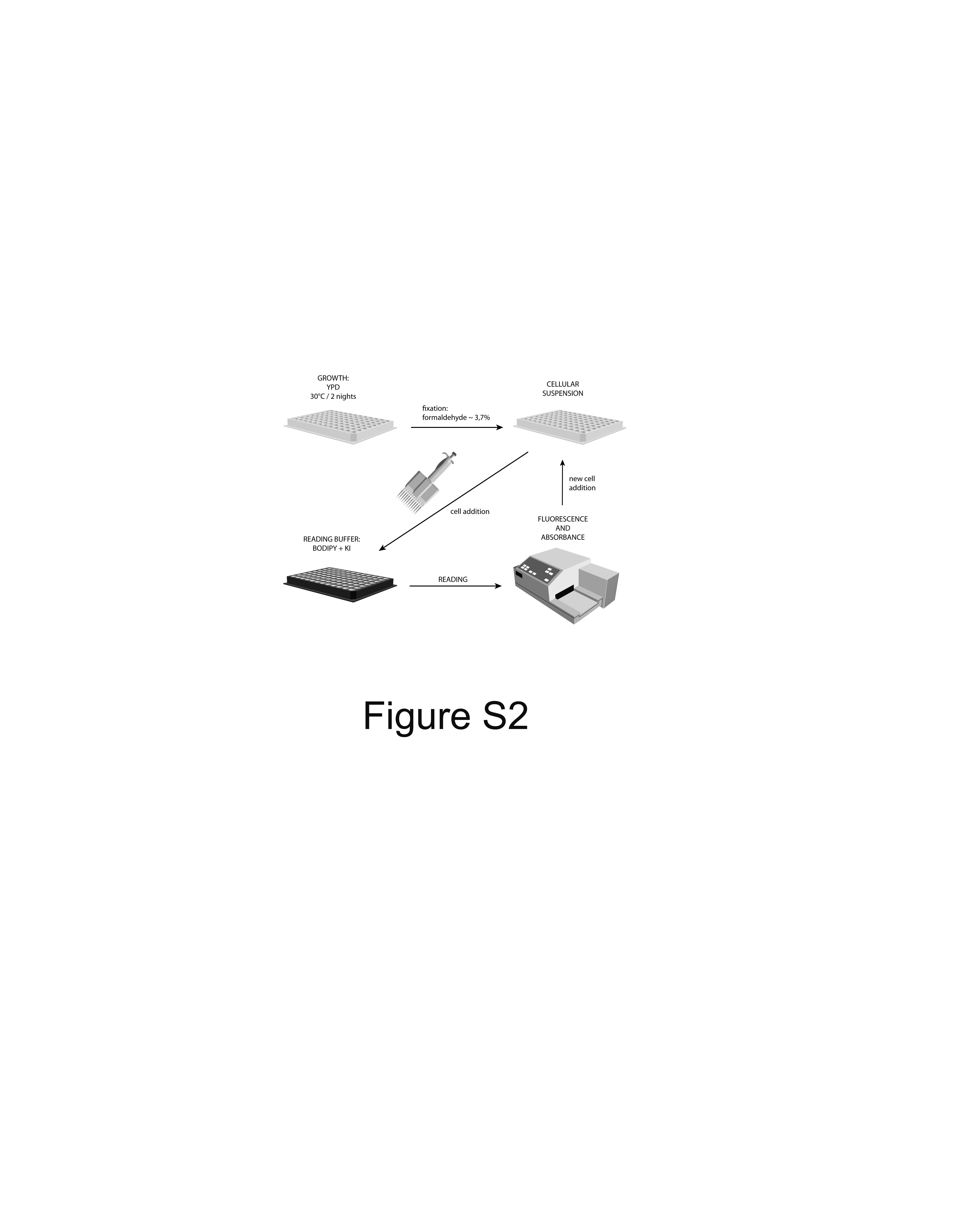

Supplement: Figure S2 — High-throughput screening of a knockout collection. Individual strains are pre-grown to stationary phase at 30°C and fixed in formaldehyde (3.7%). Cells are washed and added to a 96-well black-wall/clear-bottom plate. Fluorescence (ex/em = 485/510 nm) and absorbance (600 nm) are recorded. Three more subsequent readings were performed after the addition of cells to the wells in order to determine the LD index. (0.46 MB TIF) [file pone.0013692.s002.tif]

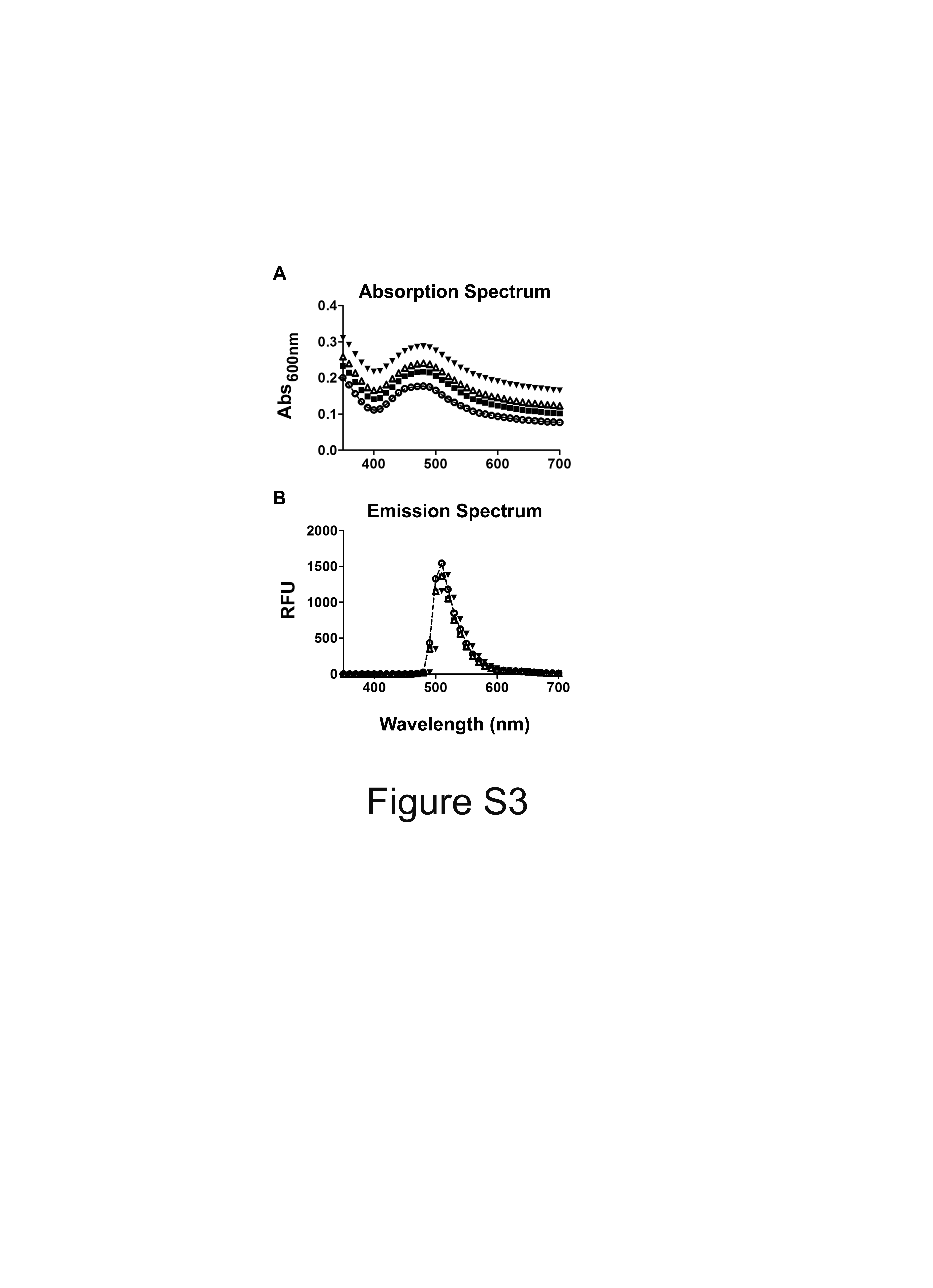

Supplement: Figure S3 — Fluorescence properties of BODIPY are not altered in the presence of cells. An aqueous solution of BODIPY (5 µM) was incubated in the presence of the indicated of none (Ο), 0.025 OD (square), 0.05 OD (Δ) and 0.1 OD (∇) of cells and the absorption spectra (upper panel) and emission (excitation at 485 nm) (lower panel) of BODIPY were recorded. Results are in agreement with information provided by the manufacturer (BODIPY Lipid Probes manual, available online at www.invitrogen.com), which states that BODIPY-derivative fluorescence parameters are insensitive to environmental conditions and not quenched by water. (0.62 MB TIF) [file pone.0013692.s003.tif]

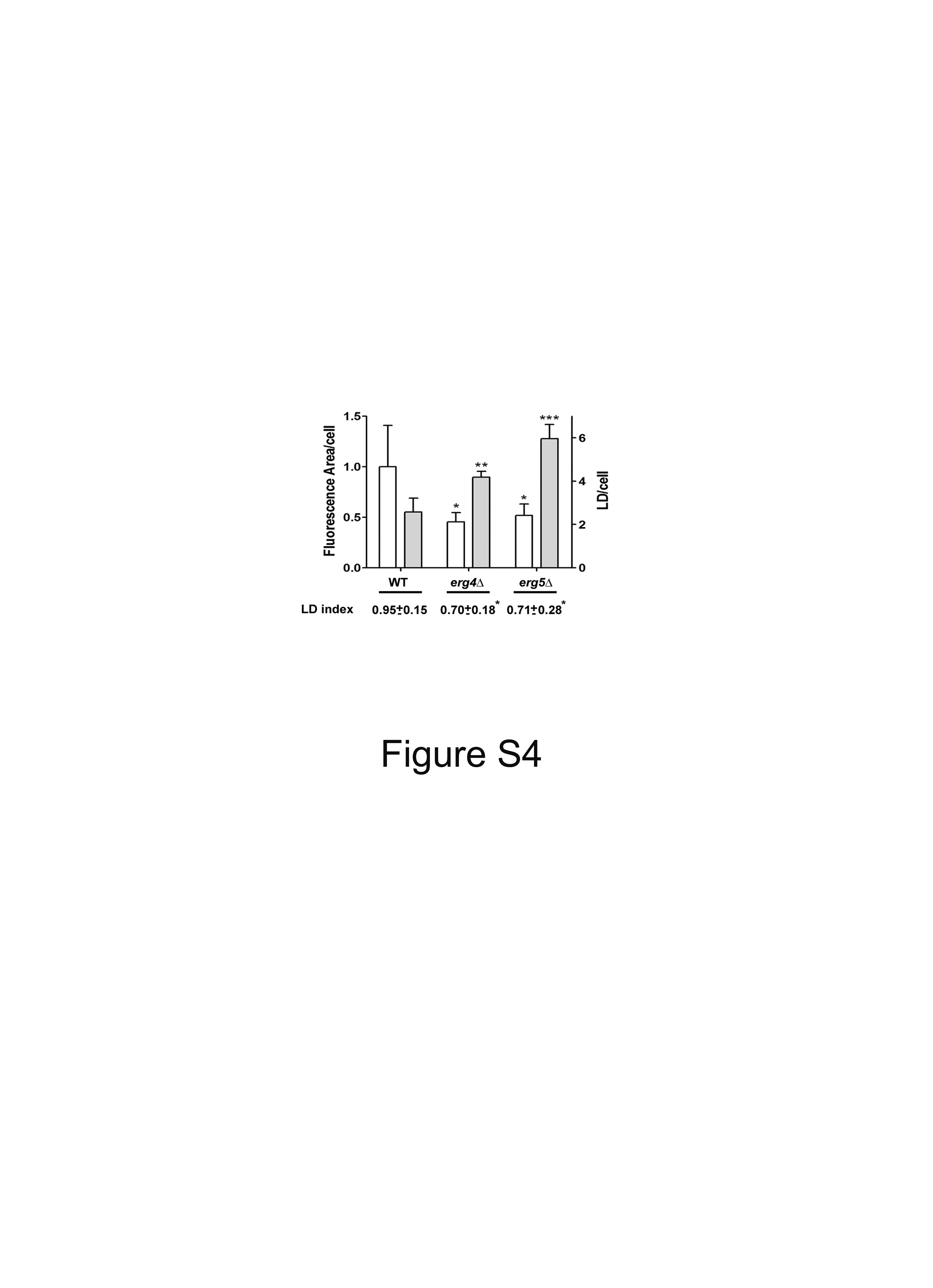

Supplement: Figure S4 — LD content in erg4 and erg5 strains was determined by fluorescence microscopy. Cells were grown to stationary phase and incubated with BODIPY. The total fluorescence area/cell was determined and expressed in pixels/cell (white bars). LDs per cell were quantified using the same images (gray bars). Data provided are for at least 100 individual cells. LD index is indicated below the graph for each strain. *p<0.05, ** p<0.01, ***p<0.001, in comparison to WT values. (1.59 MB TIF) [file pone.0013692.s004.tif]

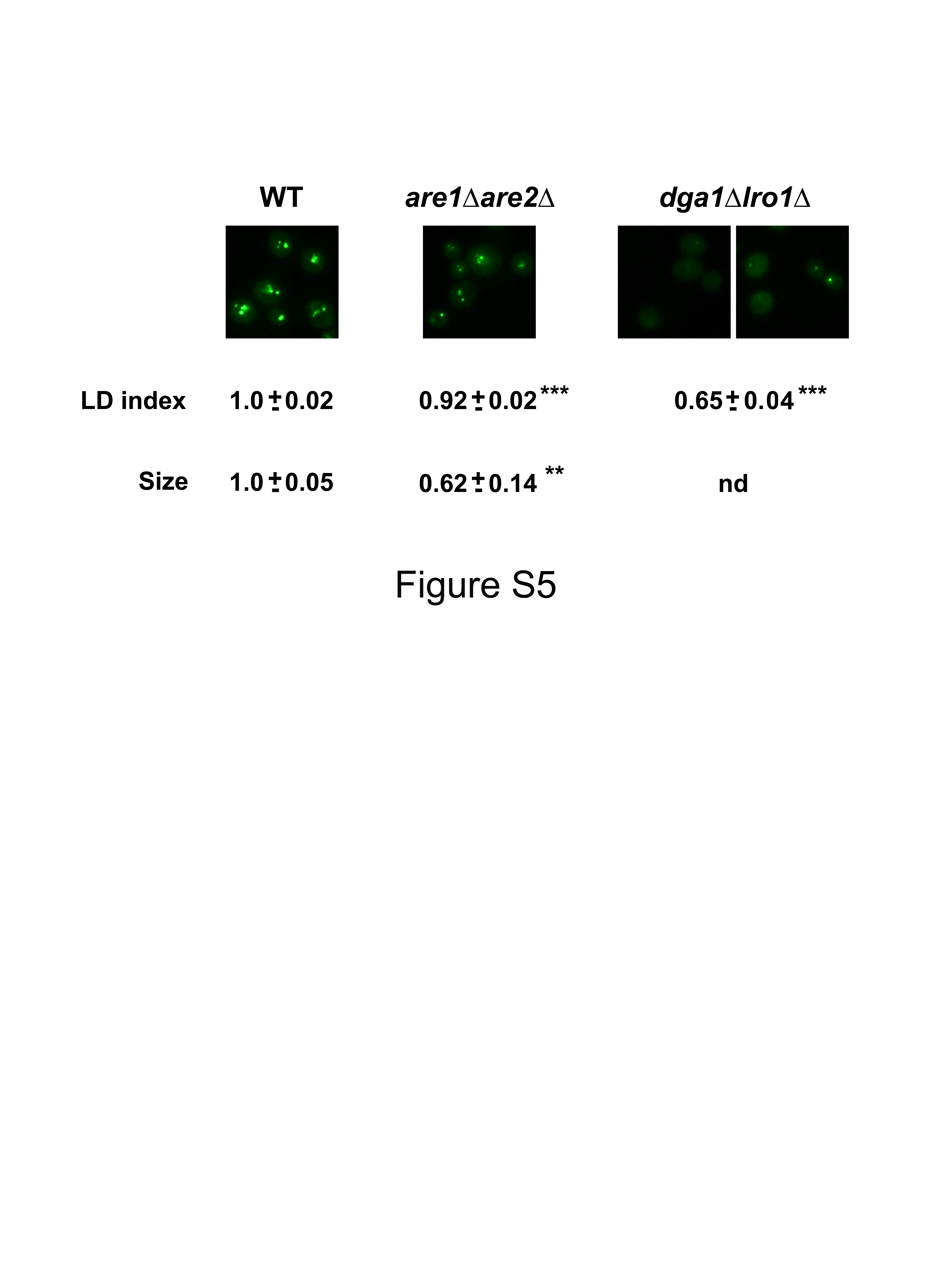

Supplement: Figure S5 — LD index correlates with either TAG or SE content. WT, are1Δ are2Δ and dga1Δ lro1Δ strains were grown to stationary phase and incubated with BODIPY. Images were captured for 17msec. A second capture (74msec) for dga1Δ lro1Δ, is also shown (last panel). At the bottom of the figure the LD index (LFR assay) (n = 3) and size (fluorescence area/cell LDs, microscopy images n = 100 cells) for each strain are shown. ** p<0.01, ***p<0.001, in comparison to WT values. (2.78 MB TIF) [file pone.0013692.s005.tif]
